# Private money-making indulgence and inefficiency of primary healthcare in Nigeria: a qualitative study of health workers’ absenteeism

**DOI:** 10.1007/s00038-020-01405-3

**Published:** 2020-08-25

**Authors:** Prince Agwu, Pamela Ogbozor, Aloysius Odii, Charles Orjiakor, Obinna Onwujekwe

**Affiliations:** 1 Department of Social Work, University of Nigeria, Nsukka, Nsukka, Nigeria; 2 Health Policy Research Group, Department of Pharmacology and Therapeutics, College of Medicine, University of Nigeria, Nsukka, Nigeria; 3 Department of Psychology, University of Nigeria, Nsukka, Nsukka, Nigeria; 4 Department of Sociology and Anthropology, University of Nigeria, Nsukka, Nsukka, Nigeria; 5 Department of Health Administration and Management, University of Nigeria, Enugu Campus, Nsukka, Nigeria; 6 Department of Psychology, Enugu State University of Science and Technology, Enugu, Nigeria

**Keywords:** Absenteeism, Primary healthcare, Informal jobs, Corruption, Market forces, Money-making activities

## Abstract

**Objectives:**

Generating additional personal income is common with primary healthcare (PHC) workforce in Nigeria, which could be because of the inconsistencies marring their monthly salaries. Therefore, this study investigates the drivers of private economic activities of PHC providers in the public sector, and the links to absenteeism, as well as inefficiency of PHC facilities in Nigeria.

**Methods:**

A qualitative study design was used to collect data from 30 key-informants using in-depth interviews. They were selected from 5 PHC facilities across three local government areas in Enugu state, south-eastern Nigeria. Data were analysed thematically, and guided by phenomenology.

**Results:**

Findings showed that majority of the health workers were involved in different private money-making activities. A main driver was inconsistencies in salaries, which makes it difficult for them to routinely meet their personal and household needs. As a result, PHC facilities were found less functional.

**Conclusions:**

Absenteeism of PHC providers can be addressed if efforts are made to close justifiable gaps that cause health workers to struggle informally. Such lesson can be instructive to low- and middle-income countries in strengthening their health systems.

## Introduction

Nigeria has a widespread of health facilities (Kress et al. 2016), and a high-density health workforce which is poorly funded by the government (Akwataghibe et al. 2013; WHO 2019). It has been found that the country regularly falls below the African Union’s (AU) 15% benchmark health funding commitment per annual budget. Since the 2001 Abuja Declaration, Nigeria has never exceeded 6% total government health expenditure per annual expenditure of government (Onyeji 2017). As a result of poor funding for the health sector, the welfare concerns of human resources for healthcare have been severely challenged, leading to steady migration of health workforce away from Nigeria, and health workers seeking additional means of income earning (Adeloye et al. 2017). Given this context, health workers are bound to miss work during official work periods leading up to a kind of absenteeism we refer to in this study as *survival absenteeism.*

There have been accounts of low prioritization of PHC facilities by most local governments, with health workers regularly owed and grossly underpaid (Adeloye et al. 2017; Oleribe et al. 2018). This is so, because, state governments through which local governments receive funding from the federal government do not specify any amount that should be spent on health, or they tend to politic with funding allocations such that local governments end up with crumbs (Kress et al. 2016). As a result, PHC providers in the public sector who are constitutionally the responsibility of the local governments in Nigeria suffer from irregular and low payments and are more quite weak in terms of staff economic welfare when compared to other health workers at the state and federal levels (Bolatito and Ibrahim 2014; Onwujekwe et al. 2019a). The resultant effect of irregular, low, or no salaries at the PHC level is that many health workers working there resort to supplementary economic activities in other to make up for their poor pay. These private economic activities in turn lead to their absence from the PHC facilities during their official work periods (Akwataghibe et al. 2013; Onwujekwe et al. 2019a). Ultimately, such cases contribute significantly to ineffectiveness and inefficiency of the public healthcare system, especially at the PHC level.

PHC facilities are dominated by the Community Health Extension Workers (CHEW) who undergo certificate training in community health extension for a period of 2–3 years (Uzochukwu et al. 2015). After the training, they tender an employment application to the local government authority who statutorily oversees the PHC in Nigeria, and supported by the National/State Primary Health Care Development Agencies (NPHCDA and SPHCDA). Upon absorption, they are deployed to PHC facilities around the local government, through the Head of Department of Health Unit in the local government. The main roles of CHEWs are in line with the statutory healthcare obligations of PHC facilities which are general preventive, rehabilitative, promotion and curative health services (Onwujekwe et al. 2019a). PHC facilities basically serve rural and hard-to-reach populations, and are the first stop for most persons in that group. When healthcare demands exceed their capacity, they make referral to secondary or tertiary health facilities. Although, some local governments hire doctors on contract to cover as much facilities they have within their remit. But due to the nature of the contract which causes them to move about, they are hardly seen for long in any PHC facility. Moreover, the hired doctors are also poorly paid by the local government, which affects their commitment (Onwujekwe et al. 2019a).

The least staff-level in PHC facilities is level 4, and persons in this group earn about N20,000 ($55) monthly, which is below the recently approved minimum wage of N30,000 ($83). Those in the highest staff-level which is level 14 earn about N95,000 ($263) (Onwujekwe et al. 2019a). Generally, health workers at the PHC level serially complain about being poorly paid amidst other shades of payment irregularities, and they are forced to assume that they are deliberately neglected by the government (Adeloye et al. 2017).

There is some existing evidence on how payment irregularities affect the effectiveness and efficiency of the health workforce in public health facilities. Akwataghibe et al. (2013) observed a huge expenditure-income disparity involving health workers from two States in Nigeria. In the study, health workers adopted informal jobs like trading and farming, and also, private practice, to even-up their expenditure-income. They earn almost or above their monthly salaries from the extra jobs they do. Also, Kress et al. (2016) consider unhealthy politics and economic constraints at the state level as central to the uncertainties marring flow of funds to local governments, which has undermined the wherewithal of local governments to effectively finance PHC facilities within their territories. The effects of these constraints on optimal health system functioning manifest in the poor attitude of health workers toward their job obligations, industrial actions and absenteeism (Adeloye et al. 2017; Oleribe et al. 2018; Onwujekwe et al. 2019a).

Absenteeism of health workers is gaining increasing attention in health system-related studies (Kisakye et al. 2016; Tweheyo et al. 2017). No doubt that absenteeism of health workers often appears in literature as a type of corruption (Kisakye et al. 2016; Onwujekwe et al. 2019a, b; Tweheyo et al. 2017). This is because, it is often viewed ideally where health workers are provided with all that is needed to work, yet they still skip work. Again, it is most often discussed in relation to patients’ care with not much attention offered to plights of health workers that could make them skip work. Conversations are beginning to happen that debate boundaries between absenteeism that should or should not be described as corruption (Belita et al. 2013; Onwujekwe et al. 2020).

Therefore, this paper provides new information on the phenomenon of engagement in private economic activities by healthcare workers at the PHC level, which reduces the effectiveness of the PHC system. The paper also explores the drivers of such behaviours and proffers possible remedies. Against the backdrop, a kind of absenteeism considered in this paper and less researched is *survival absenteeism.* It entails being absent from work for the purpose of seeking economic survival, especially where the workplace falls short of guaranteeing economic satisfaction. Going forward, it could be debated if such kind of absenteeism can be classified as corruption.

## Methods

### Study design and site

We adopted phenomenology in the design of this study, owing to the questions of phenomenon and contextual influences, we sought to answer using lived experiences of health workers in PHC facilities (Creswell 2013). Phenomenology equally entails reporting responses in thematic clusters, which we also adopted. The study was undertaken in Enugu state, southeast Nigeria. Enugu state has 17 Local Government Areas (LGAs), comprising 14 rural and 3 urban local government locations. Enugu has a population of 3.3 million people (National Population Commission 2010), which is served by about 1050 PHC facilities, with almost 35% classified as public facilities (Uzochukwu et al. 2015). The public PHC facilities happen to be the focus of this study. The state is predominantly occupied by the Igbo ethnic group who are the indigenes of southeast Nigeria, where Enugu is located.

### Sampling

Data were collected between September and October 2018 from two purposively selected sites, which were one urban and two rural local government areas. From the three local government areas, we purposively picked two facilities to represent the urban location (Enugu North) and three facilities across the rural locations [Ezeagu (1) and Nkanu East (2)]. The urban facilities were selected based on easy-to-reach, while those in the rural locations represented hard-to-reach facilities. We conveniently selected 30 participants [26 healthcare staff and 4 health facility committee chairpersons (HFCCs)] across all selected facilities to participate in the study. In all, 12 respondents were selected from the urban region, while 18 respondents came from the rural region. We had more respondents from the rural region because we picked more facilities there. On the meaning of HFCCs, they are indigenes of communities who are appointed by their traditional councils to carry out oversight functions on PHC facilities in a bid to encourage optimal performance and community ownership. Lastly, all Officers in Charge (OIC) of the selected facilities participated in the study. Since the OICs are all senior staff, in addition to other staff that had worked for 10 years and more, we automatically had a representation of senior and junior health workers.

### Data collection

A pre-test of the in-depth interview (IDI) guide was done in a different PHC facility located in Enugu North LGA. Interviews with the health workers were either conducted in the facilities or private locations, whilst the interviews with the HFCCs were conducted in their offices or residences. Signed informed consent forms were obtained from all respondents before they were interviewed using the IDI guide. All interviews were audio recorded.

### Data analysis

In line with phenomenology in qualitative research design, we generated thematic categories after responses were carefully studied (Babbie 2010; Creswell 2013). The researchers worked in pairs to enter the data into a coded MS Word Excel spreadsheet. At completion, the several data entry sheets were reviewed internally to ensure that responses were well classified, before they were harmonized. This activity complies with observer triangulation in qualitative data analysis. We also adopted peer debriefing, as two scholars from the London School of Hygiene and Tropical Medicine were handed the harmonized data entry sheet including the research questions to review. Their comments helped to further refine the analysis. Padgett (2008) advised the application of observer triangulation and peer debriefing to qualitative studies for the essence of scientific rigour.

## Results

The results are presented in four thematic categories. We begin by describing the sociodemographic features of the participants, the economic conditions of the health workers, the alternative jobs they do to survive economically which lead to their absence at the facilities, and what they think can be done to save the situation. Summarily, four themes are presented here.

### Demographic characteristics of the respondents

Table 1 shows that out of the 26 health workers that participated, more (61.5%) came from the rural location, which is as a result of the greater number of rural facilities we selected. Among the health workers, CHEWs (Community Health Extension Workers) happened to have the highest figure (42.3%). Majority (61.5%) of the health workers had above 10 years of working experience, and another majority (69.2%) had an extra engagement to supplement their income. Finally, the HFCCs were all experienced given the number of years they reported to have occupied the position.

**Table 1:** Percentage distribution of respondents. *Source*: Field survey in Enugu State, Nigeria (2018)

Sociodemographic features	Frequency	Percentage
*Health workers*		
By geographical distribution		
Urban	10	38.5
Rural	16	61.5
Total	26	100
By professional duty		
Doctor	1	3.8
Community Health Extension Worker (CHEW)	11	42.3
Midwife	8	30.8
Nurse	6	23.1
Total	26	100
By length of experience		
10 years and below	10	38.5
Above 10 years	16	61.5
Total	26	100
By involvement in additional job		
Yes	18	69.2
No	8	30.8
Total	26	100
*Health facility committee chairpersons* (*HFCCs*)		
By geographical distribution		
Urban	2	50
Rural	2	50
Total	4	100
By length of experience		
10 years and below	1	25
Above 10 years	3	75
Total	4	100

### Economic conditions of PHC workers

PHC health providers complained about the prevalence of either poor or no salaries at times, as well as delay in payments. They mentioned how the payment irregularities caused them difficulties in meeting their private welfare demands, including lacking transportation fare to come to work.

We are paid monthly, but it is always delayed. My salary does not compensate for the work I do. It kills my morale. I do not have money to even provide minor things at home, including feeding (Nurse, Ezeagu LGA).

Do you know I have stayed for up to 3 months or more without salary? I just do this work because I have a good mind. Most of the times, I borrow money to come to work and wait to pay back whenever we are remembered (more like whenever they are paid) (CHEW, Enugu North LGA).

Imagine how much time, emotions and money I must have spent studying medicine and then I graduate, only to be paid peanuts […] My superior handles 7 PHC facilities in this LGA, and he must go round all of them weekly. His salary is not enough to fuel his car (Doctor, Enugu North LGA).

Some participants made a comparison between the earnings of the health workers at the primary healthcare level and those in higher healthcare levels, signalling that theirs is poor and they are not happy about it.

You do not expect a doctor who earns about 300,000naira in a teaching hospital, and he comes here shuttling through several PHC facilities in one LGA and he is earning about 100,000naira. This is not fair at all (Doctor, Enugu North LGA).

Many participants felt that the provision of living quarters and other social amenities, as well as some other forms of job incentives, would have improved their economic conditions amidst the inconsistencies that characterize their salaries.

When my father was alive and worked here, they do get Christmas gifts from the local government, like food items and other goodies. This will make them happy and proud as government workers. But this time, we could go for the Christmas holidays without salary. It becomes difficult to buy clothes and food for the children […] It is so painful (CHEW, Ezeagu LGA).

We sometimes use our money to run the health centre. I made the table with my own money. I equally repaired that door over there when it got spoilt. These are sacrifices I make from my meagre income […] If we have a comfortable place to live around the health centre, we would not be battling transport and rent issues… (CHEW, Enugu North LGA).

These health workers are scared about their pensions. No one talks about it. Since January, those who are retired are yet to be paid their pensions. Do you want them to retire into hunger and death? That’s the reason they try to do other things (HFCC, Enugu North LGA).

### Alternative jobs and survival absenteeism

Harsh economic conditions experienced by PHC workers were found to be a reason for their absence at the PHC facilities, as they sought varying alternative and additional jobs. See quotes below:

The doctors are not paid well. They always complain that they are paid too little by the local government. So they are left with no choice than give their services to private hospitals or even try to open their own in order to help themselves (HFCC, Enugu North LGA).

It is just because of the way we are paid that we keep looking for additional jobs. This is the reason the Nigeria Labour Congress (NLC) had to strike some time ago. You can’t be earning the peanuts we earn and use that to train your children in school. Let us not even talk about when they fall sick, wear good clothes, or even eat good food. If we get good salaries, we will stay here and do the job very well. There are health workers who even ride motorcycles for commercial reasons, just so they can feed their families (Doctor, Enugu North LGA).

I have this staff who sells nylon bags at the market. You need to see how she begs us to cover for her. Her husband is out of job and she needs to survive. You can’t blame her for her absence. In fact, if you do, then you don’t have a good conscience (Nurse, Nkanu East LGA).

Here, we don’t mind leaving the facilities locked on market days. At least, I am sure that if I go to the market with the things I get from my farm, I must surely sell something and have some money to help my family. It is better than staying here and waiting to be paid at the end of the month, and finally, get disappointed (Midwife, Nkanu East LGA).

For some of the health workers, they engage some extra activities just to have food for their households to eat. They feel it helps them manage the little salaries they get or provides food for them in times when the salaries are not forthcoming. It was found that some health workers were deeply involved with the extra activities they do and might not be ready to commit fully to their primary jobs. This is very significant for those who are somewhat satisfied with the earnings they get from the extra jobs.

Sometime in the past, we were owed for up to a year by the local government. We had to start looking for other sources of income. After that period, we experienced steady salaries for a while, yet people refused to leave those extra jobs. Some found out they made more money than their monthly salaries (Nurse, Enugu North LGA).

### Suggested remedies

The respondents offered some insights on how the situation could be remedied, which included improved salaries and processes of payment, and also additional financial incentives by the government to health workers. Some key quotes are:

If our salaries can be increased from 29,000naira to at least 80,0000naira, it will go a long way. What we are paid here is too small, yet it does not come regularly. You cannot tell someone who does not have food or who cannot even transport herself to work to always be available here. It is not fair. They will rather prefer to go farming (CHEW, Nkanu East LGA).

We need incentives as they have refused to improve the salary payments. They can be giving us disinfectants, detergents, rice, or even transport allowances. We can be receiving those ones and be managing ourselves and also be happy coming to work (CHEW, Enugu North LGA).

Also, in the absence of living quarters, the participants mentioned that their postings should be made around health facilities close to where they live or transportation allowances can be made available to them.

Since we are complaining about salaries they should help us save transportation cost by building good staff quarters for us or give us transportation allowance. If that is done, we will know that we don’t need to transport ourselves to work from the small money they give us, and we will live close to the facility and attend to patients always (Nurse, Ezeagu LGA).

More so, the participants felt that high-profile members of their communities and good-spirited agencies could equally get involved in helping them.

It can be possible that some well-to-do-persons and even international and non-government organizations can help improve our welfare. It must not be the government all the time. They can decide to build staff quarters for us, add something to our stipends, do things that will increase our morale and concentration on this job. I know they can do it (Doctor, Enugu North LGA).

## Discussion

This study joins in the conversation on absenteeism and performance of the PHC facilities by considering the economic conditions of health workers, the pressures to make money, and an available market where money can be made. It establishes *survival absenteeism* to be a kind of absenteeism that entails skipping work to survive harsh economic realities that cannot be catered for by the workplace. Thus, in this context, attending work committedly makes the health worker economically vulnerable, and skipping work becomes a means of economic protection. Our study showed that same experience applied across urban and rural locations.

We engaged 26 healthcare staff and 4 HFCCs in a robust conversation in Enugu State, Nigeria. The high numbers of CHEWs that were interviewed was because CHEWs are basically trained to serve the health needs of the grass-root, and usually comprise the largest workforce in PHC. Doctors are rarely employed at the PHC level in Nigeria because of their high wages. However, some local governments might employ just a doctor who supervises all PHC facilities within their territories. The work pressure and weak incentives reinforce their perpetual unavailability. Hence, we could only interview just a doctor.

The study showed that the participants reached a consensus that they experience several payment irregularities, which include, delayed payments, half salaries, non-payment of salaries and poor pay. These irregularities were reported to have thrived for a long time. Our study falls short of empirically investigating the reasons for the persistent payment irregularities. However, in the literature, it is argued that the existing challenges of the local government structure in Nigeria which include undue political interference and weak supervision of its affairs by the federal and state governments affect the governance of PHC facilities (Bolatito and Ibrahim 2014; Kress et al. 2016).

The economic conditions of the health workers were found to be sub-optimal because of lack of finance to meet their basic needs including those of their dependents, earnings not commensurate to their work inputs, and even the lack of transportation fare to attend work. Many PHC workers hence take loans in order to survive and they are expected to pay back whenever their salaries are paid. This implies that their salaries only end up as collateral against their borrowings, forcing them the more into considering other income earning options. Some could even trek long distances to work when they are in short of money. Thus, health workers at the PHC level felt they deserve improved earnings to at least match those of their colleagues at the secondary and tertiary levels of healthcare. Similar finding exists in other studies (Akwataghibe et al. 2013; Onwujekwe et al. 2019a).

In addition, the absence of living quarters and job incentives like Christmas gifts and food items demotivate the workers and sometimes led them to spend beyond their financial capacities. The availability of living quarters within the PHC facilities would save costs in terms of rent and transportation. Also, increased government investments on the maintenance of the PHC facilities will not force the health workers into using their meagre earnings on some maintenance needs of the facilities in order to provide health services.

More so, the inference from the problems with payment of pensions after retirement is the scare of not getting their pensions by the time they retire (Oduoye 2019). This forces many health workers into considering other income earning options so they could save for the *rainy day*. Clearly, such circumstance also contributes to absenteeism from work for private income-earning activities.

We discovered that the alternative jobs undertaken by the health workers could either be formal or informal. The doctors, nurses and midwives were found more involved in private or dual practices, where they have private clinics or hospitals. Doctors, especially, could work in different facilities at the same time. Private or dual practices in the context of our study were found to be normative and barely disproved by the participants. On the part of the CHEWs, given their credentials, they resort to informal jobs like farming, trading and riding commercial motorcycles. Non-CHEWs apart from the doctors were also found involved in informal jobs. Some of the health workers got involved in farming for the purpose of household feeding. They explained that it saves the cost of buying certain foods, especially in those times when their salaries are inconsistent.

Given the roles played by the mentioned economic activities in the reinforcement of absenteeism, Onwujekwe et al. (2019b) discussed them as corruption, relying on the belief that they are done for private gains. However, our study puts forward the need for a re-think, since we observed that the absent healthcare staffs skip work because of the economic survival of themselves and their dependents. Thus, where their economic survival can be guaranteed by their primary workplace, there is the conviction that they will be present and efficient at work. The exception to this conviction should be those who argue that the extra jobs they do offer them more finance than their primary jobs, or those whose economic demands are so huge that an extra job becomes the only available solution. Nevertheless, the majority of participants suggested that improved salaries and consistencies in payment would help remedy the issues that put them away from the workplace. Other suggestions include additional incentives like living quarters, transportation allowances, posting them around facilities not far from their residences and an efficient pensioning system. They were also of the view that high-profile members of their communities and good-spirited agencies could help shoulder some of these responsibilities, as against leaving all for the government.

## Conclusion

Our study offers a somewhat novel perspective on the absenteeism of health workers, particularly in Nigeria. Cost of living in the country is increasingly on the high side, which formed a crucial reason for the labour union to make urgent demands for an upward review of wages which is yet to be implemented (Eme and Ogbochie 2017). For many Nigerians with better wages than local government employees, life is already difficult (World Poverty Clock 2019). For employees to give their best at the workplace, they need to have a secured welfare, since it is almost impossible to shut down the alternative sources of additional income. The health workers believe that their survival and those of their dependents are important to them, causing them to clamour for improved funding of primary healthcare in Nigeria. Sub-optimal funding of PHC facilities is traced to local governments who are constitutionally responsible for primary healthcare in Nigeria (Oleribe et al. 2018). However, this paper has highlighted a number of factors influencing poor funding of PHC facilities by the local governments. Some pressing ones include the general lack of prioritization of healthcare in Nigeria, the unhealthy political romance existing between state and local governments, and the lack of emphasis on primary healthcare by the central government.

Since the workplace has failed to secure economic safety for the primary healthcare workers, alternative means of survival has become needful, even if it demands that they keep facilities under *lock and key*. This is supported strongly by Homan’s (1961) rational choice theory. Thus, when absenteeism becomes *survival* there is the confusion about its description as corruption or not. Therefore, it becomes important to close up these justifiable excuses that would warrant a *humane* look at absenteeism. That way, absent health workers can be called out and dealt with accordingly. A limitation of this study is concerned with its concentration in just south-eastern Nigeria. We recommend similar studies in other geo-political zones of Nigeria and countries in the global south, so we could have a robust view on the issue.
